# Rosinidin Flavonoid Ameliorates Hyperglycemia, Lipid Pathways and Proinflammatory Cytokines in Streptozotocin-Induced Diabetic Rats

**DOI:** 10.3390/pharmaceutics14030547

**Published:** 2022-02-28

**Authors:** Sadaf Jamal Gilani, May Nasser Bin-Jumah, Fahad A. Al-Abbasi, Muhammad Shahid Nadeem, Syed Sarim Imam, Sultan Alshehri, Mohammed M. Ghoneim, Muhammad Afzal, Sami I. Alzarea, Nadeem Sayyed, Imran Kazmi

**Affiliations:** 1 Department of Basic Health Sciences, Preparatory Year, Princess Nourah Bint Abdulrahman University, Riyadh 11671, Saudi Arabia; sjglani@pnu.edu.sa; 2 Biology Department, College of Science, Princess Nourah Bint Abdulrahman University, Riyadh 11671, Saudi Arabia; mnbinjumah@pnu.edu.sa; 3 Environment and Biomaterial Unit, Health Sciences Research Center, Princess Nourah Bint Abdulrahman University, Riyadh 11671, Saudi Arabia; 4 Department of Biochemistry, Faculty of Science, King Abdulaziz University Jeddah, Jeddah 21589, Saudi Arabia; fabbasi@kau.edu.sa (F.A.A.-A.); mhalim@kau.edu.sa (M.S.N.); 5 Department of Pharmaceutics, College of Pharmacy, King Saud University, Riyadh 11451, Saudi Arabia; simam@ksu.edu.sa (S.S.I.); salshehri1@ksu.edu.sa (S.A.); 6 Department of Pharmacy Practice, College of Pharmacy, AlMaarefa University, Ad Diriyah 13713, Saudi Arabia; mghoneim@mcst.edu.sa; 7 Department of Pharmacology, College of Pharmacy, Jouf University, Aljouf, Sakaka 72341, Saudi Arabia; afzalgufran@ju.edu.sa (M.A.); samisz@ju.edu.sa (S.I.A.); 8 School of Pharmacy, Glocal University, Saharanpur 247121, India; snadeem.pharma@gmail.com

**Keywords:** rosinidin, streptozotocin, diabetes, lipid profile, proinflammatory cytokine

## Abstract

Diabetes is one of the world’s most important public health issues, impacting both public health and socioeconomic advancement; moreover, current pharmacotherapy is still insufficient. The natural flavonoid rosinidin has a long history of use in pharmaceuticals and nutritional supplements, but its role in diabetes has been unknown. The current study was intended to confirm the anti-diabetic activity of rosinidin in our laboratory setting, along with its mechanism. Streptozotocin (60 mg/kg, ip) treatment used to induce type II diabetes in rats and the test medication rosinidin was then administered orally (at doses of 10 mg/kg and 20 mg/kg) for biochemical and histopathological analysis. Treatment with rosinidin reduced negative consequences of diabetes. Rosinidin exerted a protective effect on a number of characteristics, including anti-diabetic responses (lower blood glucose, higher serum insulin and improved pancreatic function) and molecular mechanisms (favorable effects on lipid profiles, total protein, albumin, liver glycogen, proinflammatory cytokine, antioxidant and oxidative stress markers, AST, ALT and urea). Furthermore, the improved pancreatic architecture observed in tissues substantiated the favourable actions of rosinidin in STZ-induced diabetic rats.

## 1. Introduction

Diabetes mellitus is indeed one of the world’s biggest public health problems, impacting both public health and socioeconomic advancement. It has a devastating economic impact, accounting for approximately 10% of global health expenditures [1]. Diabetes mellitus is a long-lasting endocrine ailment triggered by abnormalities in the production of insulin by pancreatic cells and its actions in outlying tissues, resulting in metabolic abnormalities [2]. Despite substantial advancements in diabetes management over the last decades, patient outcomes from diabetes medications are still far from ideal.

Natural herbal plants have been a rich source of polyphenolic compounds for numerous centuries, some of which have been extensively applied to avert diabetes as a result of their medicinal qualities, multi-targeting abilities and low toxicities. Furthermore, recent studies reveal that their potential as a revolutionary medicine is promising. Several studies have suggested that some dietary bioactive substances, such as flavonoids, may influence cellular pathways [3]. Flavonoids are polyphenolic compounds having a benzo-pyrone structure. Flavonoids are plant secondary metabolites, hence they are also ingested through food [4]. Over 5000 distinct natural flavonoid compounds have been found, making flavonoids widely available. These natural chemicals have a lot of pharmacological power. This subclass was chosen since numerous studies have revealed that such compounds possess a variety of beneficial properties, such as anti-diabetic, anti-oxidant, antitumor, cardioprotective and anti-inflammatory activities, in addition to anti-cancer, anti-allergy, anti-mutagenesis, antimicrobial and antiviral activities [5]. Flavonoids’ potential to alter the insulin signaling pathway in typical target tissues such as muscle, liver and adipose tissue has been shown in abundant scientific studies to help prevent or alleviate insulin resistance in diabetics [6].

Rosinidin is a anthocyanidin flavonoid pigment found in flowers such as *Catharanthus roseus* and *Primula rosea* [7]. Rosinidin is a benzopyrylium compound with hydroxy substituents at positions 3 and 5, a methoxy substituent at position 7 and a 4-hydroxy-3-methoxyphenyl substitution at position 2. Rosinidin has a long history of use in pharmaceuticals and nutritional additions for its anti-oxidant and anti-inflammatory properties [8], all of which play prominent roles in the evolution of diabetes mellitus, yet its involvement in type II diabetes mellitus remains unknown. Molecular docking and silico target investigations demonstrated that rosinidin has the requisite structural features and has the potential to be a therapeutic molecule for neurodegenerative treatment and Parkinson’s disease neuroprotection [9].

Several new glucose-lowering synthetic medications have been approved for clinical use, each with a unique set of rewards. In this context, novel flavonoid-based compounds that favorably modify a variety of microvascular and macrovascular hazards, that function in tandem with the body’s natural defenses, and that present pharmacoeconomically favorable yet minimal side effects, are desirable. In light of this, the goal of this current study was to confirm the anti-diabetic efficacy of rosinidin in a laboratory setting. Furthermore, the impacts of these drugs on a range of targets (lipid profiles, inflammation, antioxidative-oxidant balance, as well as liver, renal and pancreatic function) were investigated in order to decipher their mechanisms. The outcomes of this study could be crucial in providing a lead for the creation of a unique flavonoid-based anti-diabetic medicine for drug companies.

## 2. Methods

### 2.1. Chemicals and Drugs for Testing

STZ (Streptozotocin) was procured from Sigma-Aldrich, St. Louis, MO, USA, analytical quality. Rosinidin (isolated compound from *Catharanthus roseus*) was obtained as a gift sample from SRL, India. Other reagents, chemicals and kits used were available locally in India.

### 2.2. Animals and STZ Paradigm

STZ’s diabetogenic action was discovered by Rakieten et al. (1963) [10]. It is especially cytotoxic to pancreatic beta-cells. Wistar rats (weighing 150–200 gm) were acclimatised for 7 days to a regular laboratory setting which included a schedule of 12-hour light and dark cycles, and standard temperature and humidity. The institute’s animal ethics committee authorized the study protocol (TRS/PT/021/006).

In rats subjected to overnight fasting, type II diabetes was produced using a 60 mg/kg STZ intraperitoneal injection prepared in a 0.1 M cold citrate buffer (pH 4.5). In order to avoid hypoglycemia-related deaths, the animals were fed a 5% glucose solution instead of water for the next 24 h. Blood samples were obtained from tail veins 48 h after STZ or vehicle injection in order to assess blood glucose levels. Diabetic rats were recognized as those with glucose levels ≥250 mg/dL, and were chosen for further pharmacological research. Rats were sacrificed at the termination of the experiment. Following termination, blood samples were obtained for various biochemical investigations and pancreases were removed and fixed in 10% buffered formalin for histopathological exploration.

### 2.3. Research Protocol Demonstration

To compare the anti-diabetic effects of the test drug rosinidin, rats were arbitrarily assigned into four experimental groups of six animals each (normal control, diabetic control, diabetic + rosinidin-10 and diabetic + rosinidin-20). The test treatment rosinidin doses (10 mg/kg and 20 mg/kg) were administered orally via a serving cannula for thirty days. While the normal control group received saline, the other treatment groups received test dosing from the first to the thirtieth day. At the beginning and completion of the study, blood samples were collected in order to determine glucose levels, and rat weights were measured.

### 2.4. Parameters

#### Changes in Body Weight

Every week, the body weights of all group animals were recorded and changes in body weight were estimated after 30 days of nourishment. At the same time, any deaths that occurred throughout the experimental period with the respective drugs were tracked.

### 2.5. Fasting Blood Glucose

To avoid the impact of homeostatic changes in blood glucose metabolism, the animals were fasted overnight and their blood glucose levels were measured at morning time. Blood samples were obtained by pricking the tips of their tails. On days 1, 4, 7 and 37, glucose was measured using a glucometer [11,12].

### 2.6. Estimation of Biochemical Parameters

The biochemical indicator evaluation was based on previously published research with slight medication. Under light anaesthesia, blood samples from all experimental rats were taken from the retro-orbital plexus at the end of study period and centrifuged in order to obtain the sera. Blood samples and homogenates were investigated in order to determine the following anti-diabetic parameters: insulin, amylase and liver glycogen. The following lipid parameters were also investigated: total cholesterol (TC), triglyceride (TG), high-density lipoprotein (HDL-C), low density lipoprotein (LDL-C), total protein and albumin. The following proinflammatory cytokines were also analysed: interleukin IL-1β and IL-6, and tumor necrosis factor (TNF)-α. The following antioxidant and oxidative stress markers were also analysed: MDA, superoxide dismutase (SOD) and catalase (CAT). Finally, the following liver and renal function markers were investigated: aspartate aminotransferase (AST), alanine amino transferase (ALT) and urea.

### 2.7. Determination of Serum Lipid and Total Protein and Albumin

Estimations of serum TC, TG, HDL-C, total protein (TP) and albumin were carried out using biochemical kits (ACCUREX, Biomedical Pvt. Ltd., Maharashtra, India). For the determination of fluorescent AGE, a 96-plate spectrophotofluorimeter was employed. The fluorescence intensity was measured at 440 nm. The Friedewald formula was used in order to compute serum LDL-C.

### 2.8. Assay of Serum Insulin

Assays were based on a sandwich ELISA kit which contained a target-specific antibody that is pre-coated in the walls of the microplate. Serum insulin was estimated using a commercial rat ELISA insulin kit (Sigma-Aldrich, St. Louis, MO, USA) as per standard provided procedure.

### 2.9. Determination of Liver Glycogen

Liver glycogen was assayed in the liver homogenate as per the anthrone reagent method [13]. In brief, 500 mg of liver tissue was homogenized in 1.5 mL of 5% potassium hydroxide and boiled for 30 min. Later, 5 mL of 95% ethanol were added to precipitate glycogen. After precipitation, test tubes were centrifuged and the supernatant decanted. Finally, the pellet was dissolved in anthrone reagent and the change in green color was measured at 620 nm.

### 2.10. Determination of α-Amylase

Amylase is an enzyme that catalyzes the hydrolysis of starch into simple sugar. Amylase concentration was estimated using a commercially available rat amylase ELISA kit using an autoanalyzer device (Meril Life Sciences Pvt. Ltd., Gujarat, India).

### 2.11. Determination of Proinflammatory Cytokines

The ELISA kit uses the sandwich-ELISA principle which includes a pre-coated antibody and microwell plate. The optical density was measured spectrophotometrically. Proinflammatory cytokines such as TNF-α, IL-1β and IL-6 were assayed using ELISA kits (Merck Millipore; Billerica, MA, USA) by following the assay protocol.

### 2.12. Determination of Lipid Peroxidation, Superoxide and Catalase

To measure antioxidant enzymes, the liver was dissected and washed in ice-cold saline, then homogenized in a 0.1 M Tris–HCl buffer (pH 7.4). The homogenate was centrifuged and supernatant was used for the assay of antioxidant enzymes, namely superoxide dismutase (SOD) and catalase (CAT). Lipid peroxidation was estimated using OxiSelect kits.

### 2.13. Determination of Aspartate Aminotransferase (AST), Alanine Aminotransferase (ALT), Urea

Colorimetric assays were used in order to assess serum concentrations of liver enzymes (AST, ALT) in addition to the renal parameter (urea) using commercially available kits according to the manufacturer’s instructions (Modern Lab., Maharashtra, India). Colorimetric analysis is based on the interaction of an enzyme with a substrate, and then to estimate colorimetrically a coloured, light-absorbing complex formed by adding another reagent after the enzyme reaction has stopped.

### 2.14. Histopathological Studies of the Pancreas

The rats were sacrificed at the termination of the experiment (37 days). Pancreatic tissues were immediately fixed in a 10% buffered neutral formalin solution. The tissues were meticulously embedded in molten paraffin using metallic blocks, then covered with flexible plastic castings and arranged under freezing plates in order to solidify the paraffin. The fixed tissues were sliced into 3–5 μm thick cross sections. In order to analyse the microscopic architecture of the pancreas, these tissue sections were stained with haematoxylin and eosin before examination under a light microscope. The sections were then observed independently by two blinded investigators.

### 2.15. Statistical Analysis

A one-way ANOVA statistical test was used to measure the differences among groups using GraphPad Prism® version 5 statistical software, San Diego, CA, USA, and *p <* 0.05 defined the statistical significance.

## 3. Results

### 3.1. Assessment of Body Weight Changes in Diabetic Rats

**Figure 1:**
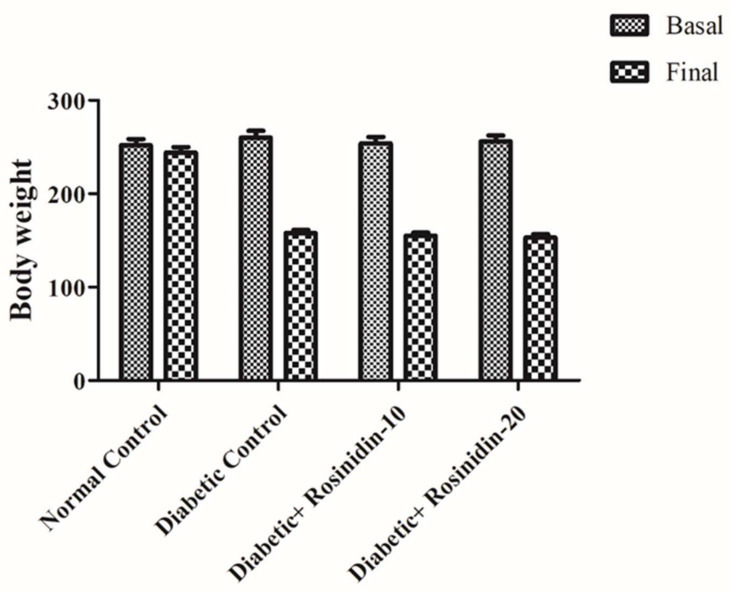
Effect of rosinidin treatments on body weights against those of STZ-induced diabetic rats (*n* = 6).

In general, diabetic rats displayed overt diabetes symptoms such as polyphagia, polydipsia and polyurea. The diabetes was proven by reduced body weight in conjunction with biochemical indicator changes (e.g., increasing blood glucose). Rats were weighed twice, at the outset of the experiment (initial weight) and again at 24 h once the last dosage of either medication was administered (final weight). When compared to normal control rats, diabetic rats exhibited a lower body weight after 30 days. The effect of rosinidin was to sustain body weight in STZ-induced diabetic rats. Figure 1 depicts no significant difference observed between treatment groups (rosinidin at different doses) as compared to diabetic control group rats.

### 3.2. Experimental Evidence for Proposed Place of Rosinidin as an Anti-diabetic Therapy

#### 3.2.1. Rosinidin Effect on Glucose in Diabetic Rats

**Figure 2:**
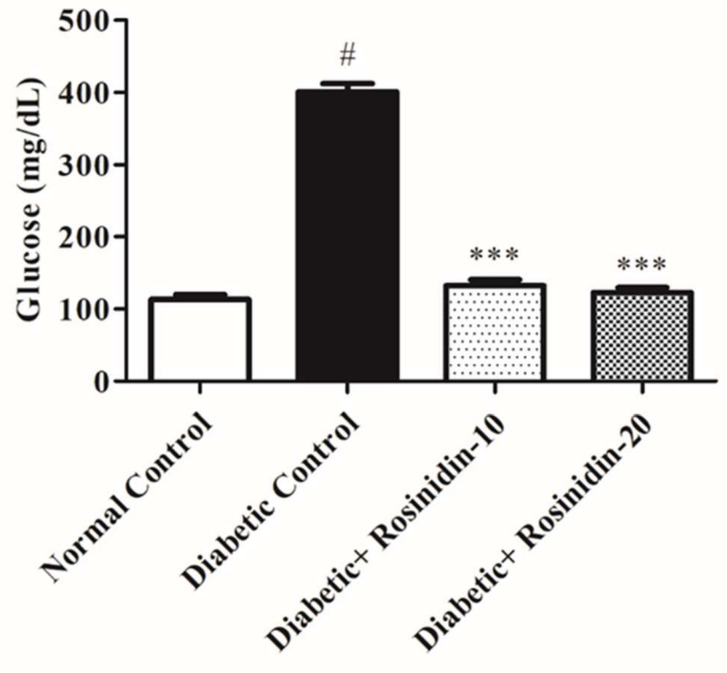
Effect of rosinidin on blood glucose against that of STZ-induced diabetic rats (*n* = 6). Values are expressed as mean ± SEM. # *p <* 0.05, normal control vs. diabetic control; *** *p <* 0.001, diabetic control vs. rosinidin-10, rosinidin-20.

Figure 2 depicts fasting blood glucose levels in rats from various experimental groups. Blood glucose levels in the diabetic control group were higher (*p* < 0.05) after STZ 60 mg/kg intraperitoneal injection than in normal animals. However, in comparison to the diabetic control group, oral administration of rosinidin 10 mg/kg and 20 mg/kg restored the hyperglycemia (*p <* 0.001).

#### 3.2.2. Rosinidin Effect on Insulin in Diabetic Rats

**Figure 3:**
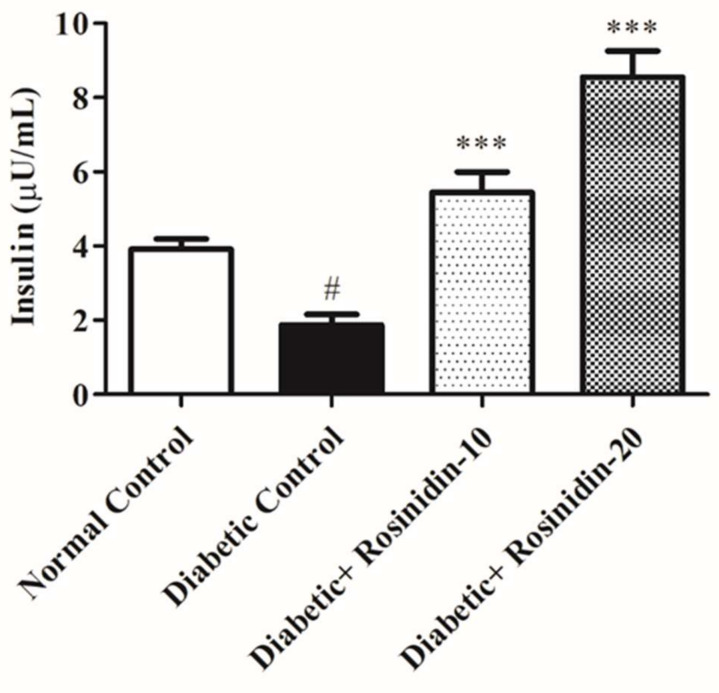
Effect of rosinidin on insulin against that of STZ-induced diabetic rats (*n* = 6). Values are expressed as mean ± SEM. # *p < 0*.05, normal control vs. diabetic control; *** *p < 0*.001, diabetic control vs. rosinidin-10, rosinidin-20.

The serum insulin levels in preventive and corrective strategies are depicted in Figure 3. In comparison to the normal group, the diabetic control group has a substantial drop (*p* < 0.05) in insulin serum levels. Both treatments with rosinidin 10 mg/kg and 20 mg/kg boosted serum insulin levels as compared to the diabetic control group (*p <* 0.001).

#### 3.2.3. Rosinidin Effect on Liver Glycogen in Diabetic Rats

**Figure 4:**
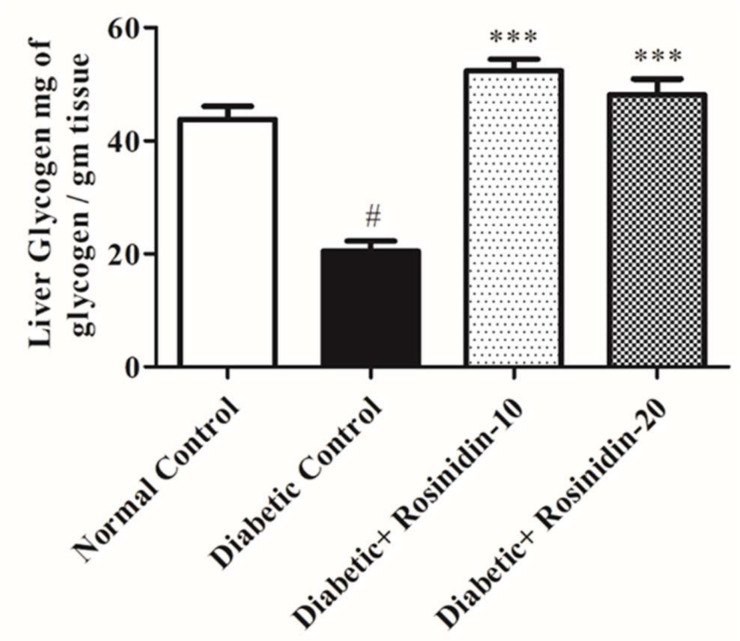
Effect of rosinidin on liver glycogen against that of STZ-induced diabetic rats (*n* = 6). Values are expressed as mean ± SEM. # *p < 0*.05, normal control vs. diabetic control; *** *p < 0*.001, diabetic control vs. rosinidin-10, rosinidin-20.

Figure 4 reveals that diabetic animals had significantly (*p* < 0.05) reduced levels of liver glycogen when compared with the normal group. The administration of rosinidin for 30 days enhanced the levels of liver glycogen in diabetic rats expressively (*p* < 0.001).

#### 3.2.4. Rosinidin Effect on Lipid Profiles in Diabetic Rats

**Figure 5:**
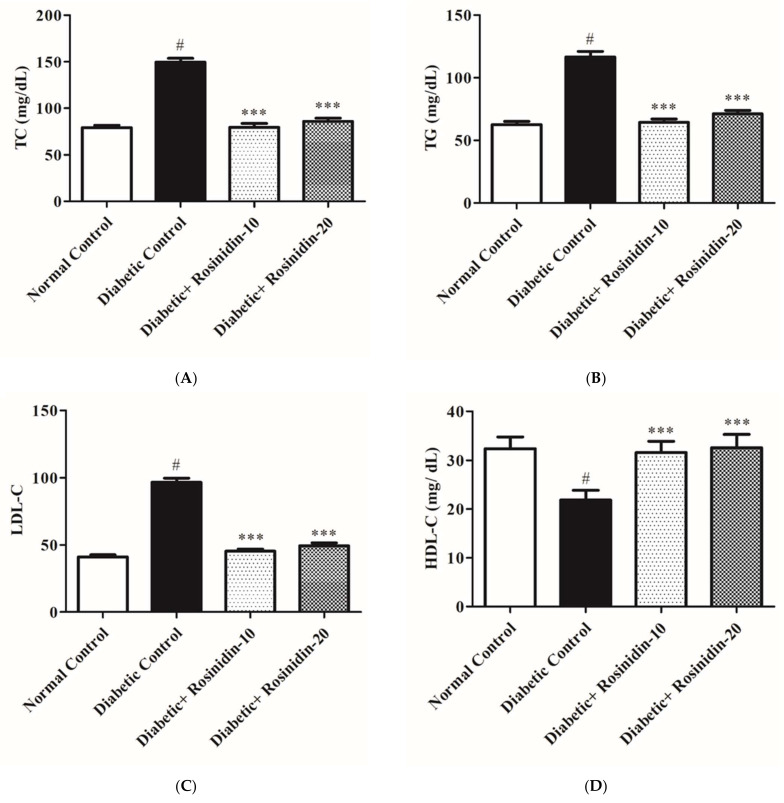
(**A**) Effect of rosinidin on TC against that of STZ-induced diabetic rats (*n* = 6). Values are expressed as mean ± SEM. # *p <* 0.05, normal control vs. diabetic control; *** *p <* 0.001, diabetic control vs. rosinidin-10, rosinidin-20. (**B**) Effect of rosinidin on TG against that of STZ-induced diabetic rats (*n* = 6). Values are expressed as mean ± SEM. # *p <* 0.05, normal control vs. diabetic control; *** *p <* 0.001, diabetic control vs. rosinidin-10, rosinidin-20. (**C**) Effect of rosinidin on LDL-C against that of STZ-induced diabetic rats (*n* = 6). Values are expressed as mean ± SEM. # *p <* 0.05, normal control vs. diabetic control; *** *p <* 0.001, diabetic control vs. rosinidin-10, rosinidin-20. (**D**) Effect of rosinidin on HDL-C against that of STZ-induced diabetic rats (*n* = 6). Values are expressed as mean ± SEM. # *p <* 0.05, normal control vs. diabetic control; *** *p <* 0.001, diabetic control vs. rosinidin-10, rosinidin-20.

Figure 5A–D depict serum levels of lipids TC, TG, LDL-C and HDL-C in various groups. When compared to normal animals, diabetic animals had a substantial (*p* < 0.05) increase in serum TG, TC and LDL-C. The Friedewald formula was used in order to compute the serum LDL-C level. The administration of rosinidin 10 mg/kg and 20 mg/kg was improved significantly (*p* < 0.001) in TC, TG and LDL-C levels of diabetic animals. In addition, diabetic rats revealed a significant (*p* < 0.05) drop in serum HDL-C levels which was prominently increased after receiving rosinidin at both dosages when compared to diabetic control rats. The administration of rosinidin at doses of 10 mg/kg and 20 mg/kg resulted in very marked improvements in all indices of altered lipid contours.

#### 3.2.5. Rosinidin Effect on Total Protein and Albumin in Diabetic Rats

**Figure 6:**
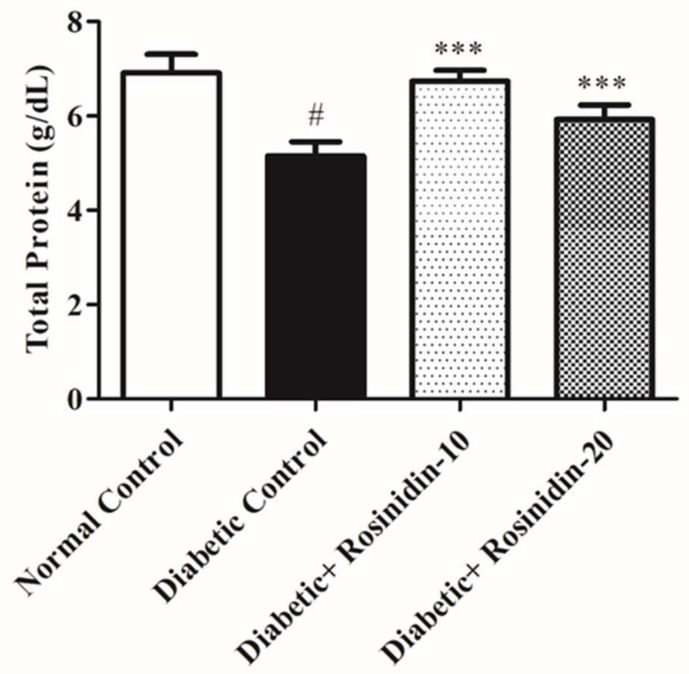
Effect of rosinidin on total protein against that of STZ-induced diabetic rats (*n* = 6). Values are expressed as mean ± SEM. # *p <* 0.05, normal control vs. diabetic control; *** *p <* 0.001, diabetic control vs. rosinidin-10, rosinidin-20.

**Figure 7:**
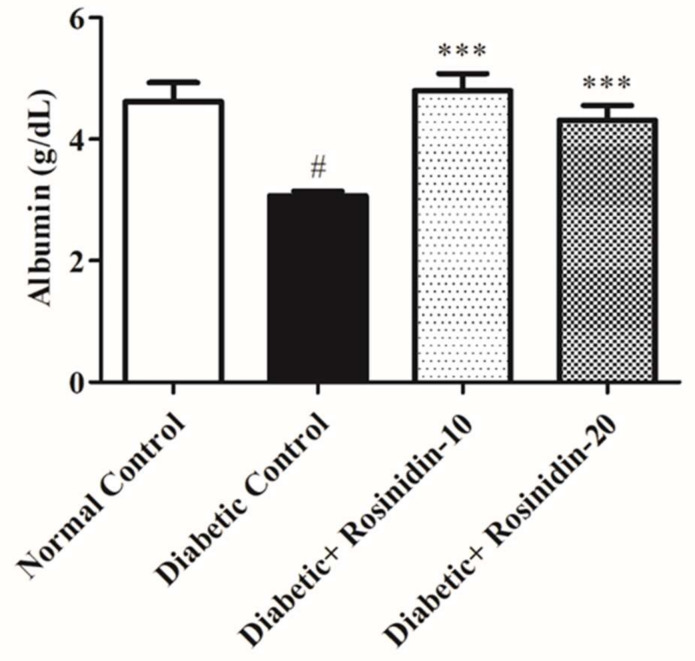
Effect of rosinidin on albumin against that of STZ-induced diabetic rats (*n* = 6). Values are expressed as mean ± SEM. # *p <* 0.05, normal control vs. diabetic control; *** *p <* 0.001, diabetic control vs. rosinidin-10, rosinidin-20.

In serum levels of total protein, albumin represents the condition of protein absorption and metabolism. The total protein and albumin levels of STZ-induced diabetic rats were considerably lower than those of the normal group rats. In the treatment group rats, rosinidin administration dramatically improved total protein and albumin levels as shown in Figure 6 and Figure 7.

#### 3.2.6. Rosinidin Effect on Proinflammatory Cytokines in Diabetic Rats

**Figure 8:**
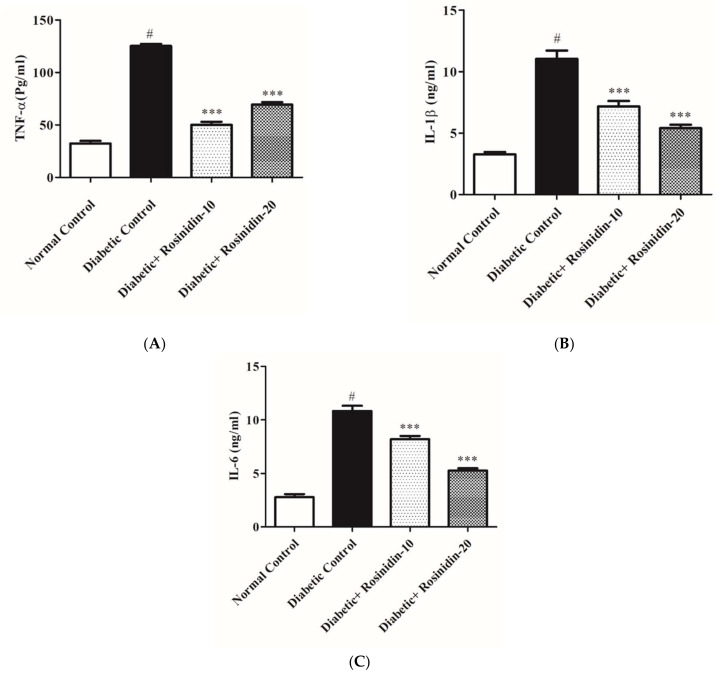
(**A**) Effect of rosinidin on TNF-α against that of STZ-induced diabetic rats (*n* = 6). Values are expressed as mean ± SEM. # *p <* 0.05, normal control vs. diabetic control; *** *p* < 0.001, diabetic control vs. rosinidin-10, rosinidin-20. (**B**) Effect of rosinidin on IL-1β against that of STZ-induced diabetic rats (*n* = 6). Values are expressed as mean ± SEM. # *p <* 0.05, normal control vs. diabetic control; *** *p <* 0.001, diabetic control vs. rosinidin-10, rosinidin-20. (**C**) Effect of rosinidin on IL-6 against that of STZ-induced diabetic rats (*n* = 6). Values are expressed as mean ± SEM. # *p <* 0.05, normal control vs. diabetic control; *** *p <* 0.001, diabetic control vs. rosinidin-10, rosinidin-20.

Diabetes induction resulted in an increase in proinflammatory parameters TNF-α, IL-1β and IL-6, which were estimated using an ELISA technique in this study. Figure 8A–C showed that STZ administration drastically increased the production of proinflammatory markers when compared to normal control rats (*p <* 0.05). Meanwhile, rosinidin therapy significantly brought down the levels of inflammatory mediators when compared to the diabetic control group (*p* < 0.001). Rosinidin implantation exerts protective effects via the inhibition of proinflammatory cytokines (Figure 8A–C).

#### 3.2.7. Rosinidin Effects on Antioxidant and Oxidative Stress Markers in Diabetic Rats

**Figure 9:**
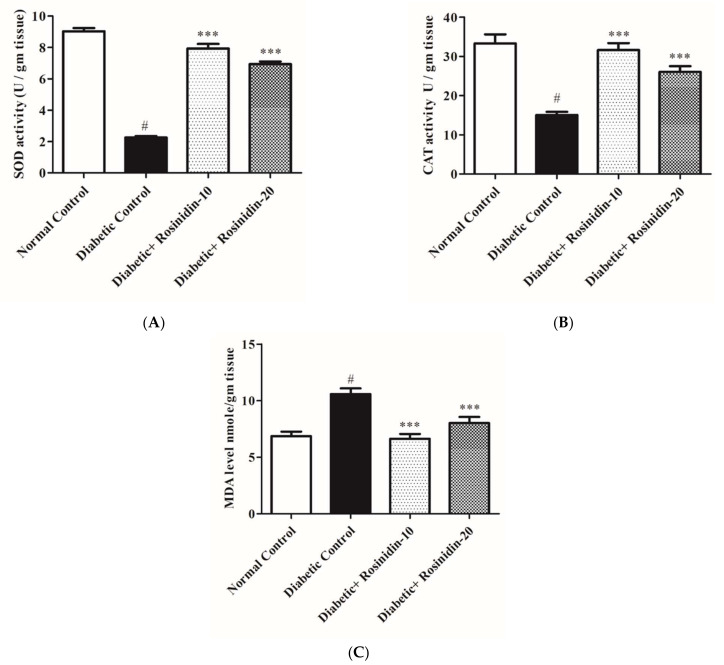
(**A**) Effect of rosinidin on SOD against that of STZ-induced diabetic rats (*n* = 6). Values are expressed as mean ± SEM. # *p <* 0.05, normal control vs. diabetic control; *** *p* < 0.001, diabetic control vs. rosinidin-10, rosinidin-20. (**B**) Effect of rosinidin on CAT against that of STZ-induced diabetic rats (*n* = 6). Values are expressed as mean ± SEM. # *p <* 0.05, normal control vs. diabetic control; *** *p* < 0.001, diabetic control vs. rosinidin-10, rosinidin-20. (**C**) Effect of rosinidin on MDA against that of STZ-induced diabetic rats (*n* = 6). Values are expressed as mean ± SEM. # *p <* 0.05, normal control vs. diabetic control; *** *p <* 0.001, diabetic control vs. rosinidin-10, rosinidin-20.

Figure 9A–C illustrate changes in lipid peroxidation and antioxidant defense system indicators such as MDA, SOD and CAT activity in experimental rats. In diabetic control rats, SOD and CAT activities were severely decreased, yet there was a significant increase in MDA levels when compared to normal animals (*p <* 0.05). Increased levels of SOD and CAT in addition to lower levels of MDA in the treatment group were shown to have a substantial upgrading in the activities of these enzymes following rosinidin 10 mg/kg and 20 mg/kg treatments as compared to diabetic control animals (*p* < 0.001). Treatment with rosinidin improved exacerbated alterations in oxidative stress markers and antioxidant enzyme activities in diabetic rats.

#### 3.2.8. Rosinidin Effect on Hepatic and Renal Function in Diabetic Rats

**Figure 10:**
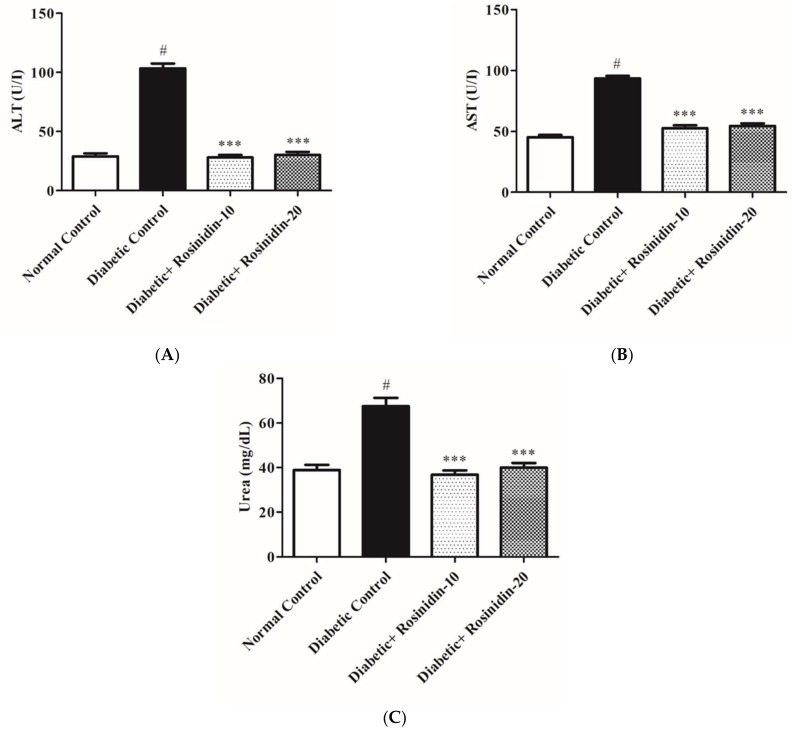
(**A**) Effect of rosinidin on ALT against that of STZ-induced diabetic rats (*n* = 6). Values are expressed as mean ± SEM. # *p* < 0.05, normal control vs. diabetic control; *** *p <* 0.001, diabetic control vs. rosinidin-10, rosinidin-20. (**B**) Effect of rosinidin on AST against that of STZ-induced diabetic rats (*n* = 6). Values are expressed as mean ± SEM. # *p* < 0.05, normal control vs. diabetic control; *** *p* < 0.001, diabetic control vs. rosinidin-10, rosinidin-20. (**C**) Effect of rosinidin on urea against that of STZ-induced diabetic rats (*n* = 6). Values are expressed as mean ± SEM. # *p <* 0.05, normal control vs. diabetic control; *** *p <* 0.001, diabetic control vs. rosinidin-10, rosinidin-20.

When diabetic control rats were compared to the normal group, serum biomarkers ALT, AST and urea levels increased significantly (*p <* 0.05). Administering rosinidin doses at 10 mg/kg and 20 mg/kg, on the other hand, resulted in noteworthy declines in the elevated levels (*p* < 0.001) of these hepatic and renal functions, as shown in Figure 10A–C.

#### 3.2.9. Rosinidin Effect on α-Amylase Level in Diabetic Rats

**Figure 11:**
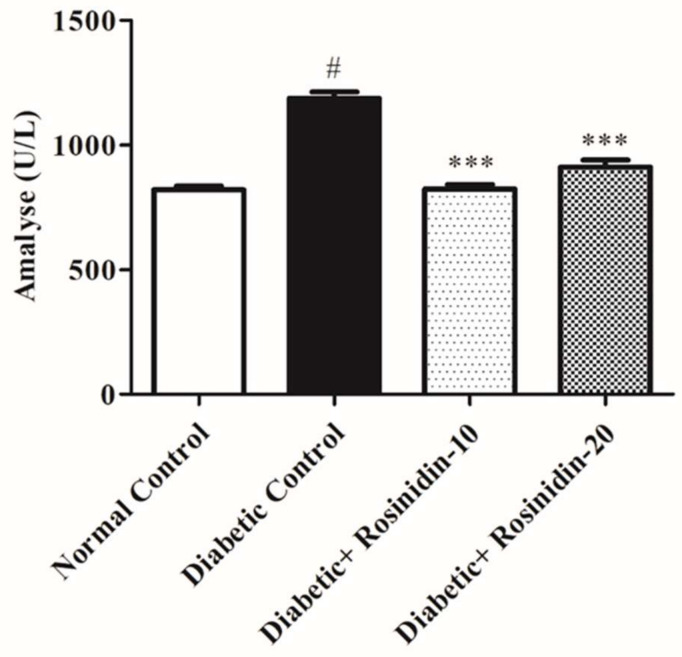
Effect of rosinidin on α-amylase against that of STZ-induced diabetic rats (*n* = 6). Values are expressed as mean ± SEM. # *p* < 0.05, normal control vs. diabetic control; *** *p* < 0.001, diabetic control vs. rosinidin-10, rosinidin-20.

Regarding α-amylase levels, a significant elevation (*p <* 0.05) in its levels was noted in diabetic rats compared to the normal rats; meanwhile, diabetic rats treated with rosinidin doses at 10 mg/kg and 20 mg/kg exhibited a substantial decrease (*p <* 0.001) in serum amylase levels compared to the diabetic rats (Figure 11).

#### 3.2.10. Rosinidin Effect on Pancreatic Function and Photomicrographs of Pancreatic Tissue

**Figure 12:**
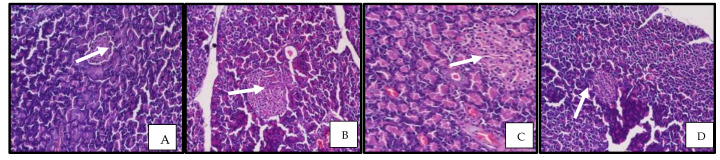
Representative photomicrographs illlustrating pancreatic tissue sections stained with H and E among various experimental groups. (**A**) Normal control: organized pattern and normal architecture of islet cell. (**B**) Diabetic control: damaged islets of Langerhans and degeneration in the pancreas portion. (**C**) Rosinidin-10: improved pancreatic histology, but minor pancreatic cell congestion. (**D**) Rosinidin-20: marked improvement in pancreatic histology. Arrows indicate islets of Langerhans.

In order to investigate therapeutic effects of rosinidin on the improvement of pancreatic performance in the STZ-induced diabetic model, histopathological modifications of the pancreas were measured. In control rats the pancreatic architecture was observed to be normal, with intact exocrine tissue and islet cells enclosed by a delicate capsule. Diabetic animals showed considerable degeneration in the pancreas portion, according to scientific evaluations. Rosinidin at the 10 mg/kg dose resulted in pancreatic histology that was analogous to that of the control rats, but with slight pancreatic cell crowding or occlusion. The rosinidin 20 mg/kg treatment group, on the other hand, showed pancreatic cell regeneration to a level similar to that of a normal rat pancreas (Figure 12).

## 4. Discussion

The problems associated with insulin and oral hypoglycemic medications have sparked interest in finding natural substances with anti-diabetic properties. As a result, safer and more effective anti-diabetic medicines in the form of flavonoid-based regimens are required. Moreover, numerous studies demonstrating flavonoids’ salutary effects on metabolic abnormalities such as diabetes mellitus and obesity have piqued scientists’ interest in flavonoids in recent decades [14]. However, the anti-diabetic action of rosinidin in STZ-induced diabetic rats has yet to be investigated. The goal of this research study was to learn more about the processes by which rosinidin protects against type II diabetes mellitus, in order to offer fresh light on type II diabetes mellitus prevention using flavonoid-based alternatives. Rosinidin at 10 mg/kg and 20 mg/kg doses ameliorated hyperglycemia, lipid pathways and proinflammatory cytokines, in addition to antioxidant and oxidative stress markers in STZ-induced diabetic rats. In addition, improvements to liver, renal and pancreatic function were also reported.

STZ is transported into pancreatic cells by the glucose transporter GLUT2. The nitrosourea moiety of this chemical alkylates DNA, which is the primary basis of STZ-induced cell loss. ROS generation, on the other hand, may cause DNA damage and other STZ-related adverse effects [15]. In the current study, STZ resulted in significant hyperglycemia, hypoinsulinemia and significant proinflammatory cytokinin, in addition to antioxidant and oxidative stress with a noticeable upsurge in levels of TC, TG, LDL-C, amylase, ALT, AST and urea; meanwhile, HDL-C, total protein, albumin and liver glycogen markedly decreased. A variety of studies have provided equivalent results [16].

The biological actions of these naturally occurring flavonoids and their metabolites provide cells with a variety of health-promoting benefits. Flavonoids can also interact directly with proteins such as strategic cellular receptors or signaling pathways, affecting a wide range of functions in many cells and tissues [17]. As a result, flavonoids can diminish insulin resistance in insulin-sensitive tissues in a variety of ways, including via modulating the insulin signaling pathway. An outcome in treating diabetic rats with rosinidin at 10 mg/kg and 20 mg/kg doses was a significant decline in blood glucose. This finding was in line with prior research conducted on flavonoids. Anthocyanins have been presented to have anti-obesity and anti-diabetic properties in a variety of animal models [18]. From in vitro and in vivo investigations, many flavonoid components, particularly auxiliaries, have demonstrated hypoglycemic effects. Apigenin, baicalein and catechin flavonoids reduce blood glucose. Hesperidin is beneficial for diabetic neuropathy, while glycyrrhiza flavonoid has a substantial effect on gestational diabetes mellitus. Some compounds, such as kaempferol and puerarin, are also favorable to cardiomyopathy [19].

Flavonoids help people with diabetes mellitus in a variety of ways. The most prevalent symptom is a decrease in glucose absorption. Flavonoids also function by reducing the action of the enzyme glucosidase in the small intestine. When utilized to explore their functions in glycoside absorption and metabolism, galangin, kaempferol and luteolin demonstrated α-glucosidase inhibitory action for both in vivo and in vitro circumstances [20]. As a result, the flavonoid rosinidin’s potential to enhance plasma insulin concentration, as established in this work, could offer a foundation for its hypoglycemic activity in diabetic rats. This outcome is in line with expectations [21]. The capacity of rosinidin to preserve body weight loss is a result of an escalation in insulin secretion and subsequent hypoglycemia reaction. Diabetes impairs glucose production in the liver and skeletal muscles of rats. The diabetic animals in this study had significantly lower hepatic glycogen levels, which were proportional to insulin insufficiency. Liver glycogen content of diabetic rats was considerably raised by rosinidin, possibly resulting from enhanced insulin output. These findings are consistent with earlier research. Li C. et al. demonstrated the instability of the diabetic hepatic glycogen structure [22].

Dyslipidemia is well-thought-out as the fundamental module of metabolic disorder that is characterized by increased levels of TC, TG and LDL-C, in addition to reduced levels of HDL [23]. The analysis of lipid profile levels in this study specified a promising effect of rosinidin for both dosage regimens. These results are in partial agreement with previous results [24]. Flavonoids are also regarded as safe for use in the treatment of diabetes-related lipid abnormalities as well as other metabolic illnesses, according to clinical evidence [25]. Furthermore, rosinidin administration resulted in an increase in total protein and albumin levels, which contributed to the test drug’s sound hypoglycemic effects.

Another important point to consider is the substantial rise in proinflammatory cytokines TNF-α, IL-1β and IL-6 in diabetic animals. Excess free radical production promotes the triggering of nuclear factor-kappa B, which in turn promotes the transcription of genes encrypting inflammatory proteins. Previously, it was reported that STZ-induced diabetes in rats promoted NF-B-mediated inflammation [26]. Rosinidin, on the other hand, was effective in lowering TNF-α, IL-1β and IL-6 in diabetic rats, implying that rosinidin may possibly play a defensive starring role against inflammatory responses in diabetic rats. Previous reports revealed similar findings [27].

The progression of diabetes, as well as diabetes-related problems, can be attributed to oxidative stress. Our study disclosed striking elevations in antioxidant enzymes (SOD, CAT) and lipid peroxidation by reduced levels of MDA, which specify the antioxidant strength of the flavonoid found in rosinidin. Rutin, luteolin and quercetin flavonoids employed patent antioxidant events that protected liver and kidney tissues against various damages, consistent with other investigations [28].

Furthermore, the diabetic group in this study had significant increases in ALT, AST and urea levels in their blood. Impairment of hepatic cells is indicated by the leaking of hepatic enzymes from the hepatic cytosol into circulation. The elevated levels of serum urea in diabetic rats suggested reduced renal function, since urea is a sensitive marker for kidney injury. Treatment of diabetic rats with rosinidin, on the other hand, resulted in substantial declines in the actions of these enzymes in addition to decreases in urea, indicating that rosinidin has a hepatorenal protective effect. These findings are consistent with prior research that found flavonoids to have hepatorenal protective properties [29]. Rosinidin molecules significantly suppress amylase enzymes, signifying anti-diabetic effects; this is consistent with prior studies on other flavonoids [30,31,32]. Furthermore, rosinidin protects vital tissues including the pancreas, thereby lowering risks of diabetes-related complications. Histological considerations of the pancreas revealed substantial abnormalities in untreated diabetic pancreatic tissue and demonstrated degeneration at the level of the islets of Langerhans when compared to healthy rats. These conclusions imply that rosinidin’s anti-diabetic properties are linked to its ability to improve pancreatic histological properties.

## 5. Conclusions

Flavonoid-based therapies could be a new target for researchers. The flavonoid rosinidin was found to control hyperglycemia and lipid metabolism, and moreover restored proinflammatory cytokine levels in STZ-induced diabetic animals. As a consequence, rosinidin’s anti-diabetic properties appear to be linked to its ability to achieve improvements in pancreatic histology. More research is desired to confirm these findings and to determine other salutary effects of rosinidin, a flavonoid-based medication, in standard anti-diabetic therapy.
